# High-fidelity scaling relationships for determining dissipative particle dynamics parameters from atomistic molecular dynamics simulations of polymeric liquids

**DOI:** 10.1038/s41598-020-61374-8

**Published:** 2020-03-10

**Authors:** M. H. Nafar Sefiddashti, M. Boudaghi-Khajehnobar, B. J. Edwards, B. Khomami

**Affiliations:** Materials Research and Innovation Laboratory, Chemical and Biomolecular Engineering, University of Tennessee, Knoxville, 37996 USA

**Keywords:** Chemical engineering, Polymers, Rheology, Fluid dynamics

## Abstract

An optimized Dissipative Particle Dynamics (DPD) model with simple scaling rules was developed for simulating entangled linear polyethylene melts. The scaling method, which can be used for mapping dimensionless (reduced units) DPD simulation data to physical units, was based on scaling factors for three fundamental physical units; namely, length, time, and viscosity. The scaling factors were obtained as ratios of equilibrium Molecular Dynamics (MD) simulation data in physical units and equivalent DPD simulation data for relevant quantities. Specifically, the time scaling factor was determined as the ratio of longest relaxation times, the length scaling factor was obtained as the ratio of the equilibrium end-to-end distances, and the viscosity scaling factor was calculated as the ratio of zero-shear viscosities, each as obtained from the MD (in physical units) and DPD (reduced units) simulations. The scaling method was verified for three MD/DPD model liquid pairs under several different nonequilibrium conditions, including transient and steady-state simple shear and planar elongational flows. Comparison of the MD simulation results with those of the scaled DPD simulations revealed that the optimized DPD model, expressed in terms of the proposed scaling method, successfully reproduced the computationally expensive MD results using relatively cheaper DPD simulations.

## Introduction

Accurate modeling and simulation of flow-microstructure coupling in entangled polymeric fluids is of great technological and scientific interest. Despite notable successes of advanced reptation (tube) based models under low to moderate flow conditions^1–5^, a number of key theoretical concepts used in these models have only been explicitly defined under quiescent conditions. How these basic concepts, such as the tube radius, number of entanglements, primitive path length, tube stretch, tube orientation tensor, etc., are extrapolated to high strain rate flows is a matter of great interest to practitioners of polymer fluid mechanics and rheology.

To address these and other relevant questions, single molecular visualization experiments are paving the way for the molecular level understanding of the complex flow behavior of entangled polymeric fluids^6,7^. Although these experiments have shed light on the complex relaxation behavior of entangled polymeric fluids, namely, polymer chain and tube relaxation times, they have yet to provide a clear molecular mechanism for the dynamics of the entanglement network topology; *i.e*., reversible flow-induced disentanglement and its effect on the macroscopic response of the fluid undergoing flow. The primary limitation of current single-molecule visualization experiments is the short time (compared to the longest relaxation time of the fluid) and the small number of molecules that can be effectively tracked simultaneously in dense entangled fluids under flow. Hence there has been a growing body of work aimed at atomistic or coarse-grained simulations of polymeric fluids. These include, atomistic Non-Equilibrium Molecular Dynamics (NEMD)^8–12^, coarse-grained NEMD^13–16^, Dissipative Particle Dynamics (DPD)^17–25^, and Slip-Link (SL) and Slip-Spring (SS) simulations^26–29^.

To date, NEMD simulations have provided a wealth of information regarding single chain dynamics and their relationship to the macroscopic rheological and microstructural properties of this class of fluids. Chief among them are (1) a molecular description of convected constraint release (CCR) including new modes of chain relaxation, and (2) entanglement network dynamics and its inherent relation with tube stretch and orientation, and hence polymeric stress^9,30– 33^. A key advantage of this “virtual experimentation” over conventional experimentation is that every atomic constituent of each molecule can be tracked at arbitrarily small time increments, thus providing a complete description of the dynamical state of the entire fluid system. Furthermore, virtual experimentation can be conducted on pure systems (*e.g*., strictly monodisperse, linear macromolecular melts) under ideal conditions without regard to instrument compliance or inertia. NEMD simulations also provide rheological, optical, and spectroscopic data in physical units that can be directly compared with experimental data^34–38^, in contrast to simulations of coarse-grained models (as discussed below). The primary limitations on what can be achieved are (1) the accuracy of the atomic potential model of the polymeric liquid and (2) the vast computational resources necessary to simulate these highly entangled macromolecular systems possessing up to 10^8^ degrees of freedom which must be tracked over multiple disengagement times.

Coarse-grained models of atomistic liquids offer a more computationally tractable alternative to brute force MD simulations wherein individual atomistic molecular units are grouped together and treated as single entities, thus greatly reducing the number of degrees of freedom to be tracked during the time integration. Mesoscale simulation methods, such as SL, SS, and DPD, have contributed significantly to the understanding of linear and nonlinear rheology of entangled fluids at length and time scales beyond the computational limitations of MD simulations. However, the accuracy of the predictions made by the SL and SS techniques strongly depends on the assumed constraint release/renewal frequency, particularly at large deformation rates. On the other hand, the accuracy of DPD simulations is directly related to how accurately various static and dynamic properties of the chain (as determined via atomistic simulations) can be used to determine the time and length scales of the associated multiatomic particles of the DPD simulations. To this end, attention is focused on developing an accurate yet simple method to obtain rescaling parameters such that DPD and MD simulation results are fully consistent over a wide range of deformation rates in common flow situations, such as steady-state and startup of shear and elongational flows.

## Methodology

Dissipative particle dynamics (DPD) was originally introduced by Hoogerbrugge and Koelman^39,40^. In this model, three types of force are applied to each particle: a conservative force derived from a potential exerted on particle pairs, a dissipative force, and a random force. The original model of Hoogerbrugge and Koelman, however, lacked an expression relating the system temperature and the model parameters. Español and Warren^41^ derived the stochastic differential equations and the corresponding Fokker-Planck equation for DPD. The temperature in this formulation was related to the random force via the fluctuation-dissipation theorem.

The forces typically employed in state-of-the-art DPD simulation of polymeric chain systems are

1 $${{\bf{F}}}_{i}={\sum }_{j\ne i}({{\bf{F}}}_{ij}^{c}+{{\bf{F}}}_{ij}^{D}+{{\bf{F}}}_{ij}^{R}),$$

2 $${{\bf{F}}}_{ij}^{c}={a}_{ij}\left(1-\frac{{r}_{ij}}{{r}_{c}}\right){\widehat{{\bf{r}}}}_{ij},$$

3 $${{\bf{F}}}_{ij}^{D}=-\gamma {\omega }^{D}({r}_{ij})[({{\bf{v}}}_{i}-{{\bf{v}}}_{j}).{\widehat{{\bf{r}}}}_{ij}]{\widehat{{\bf{r}}}}_{ij},$$

4 $${{\bf{F}}}_{ij}^{R}=\sigma {\omega }^{R}({r}_{ij})\zeta {\widehat{{\bf{r}}}}_{ij}$$

where indices represent various particles, ${{\bf{F}}}_{ij}^{c}$, ${{\bf{F}}}_{ij}^{D}$, and ${{\bf{F}}}_{ij}^{R}$ are the conservative, dissipative, and random forces between particles *i* and *j*, and **F**_*i*_ is the total acting force on particle *i*. The conservative force is a purely repulsive pairwise interaction with a repulsion constant, *a*, and a cutoff distance, *r*_*c*_. The distance between the particle pairs is represented as *r*_*ij*_, and ${\widehat{{\bf{r}}}}_{ij}={{\bf{r}}}_{ij}$/*r*_*ij*_ is the unit connector vector between the particles *i* and *j*. The dissipative force is expressed in terms of the velocities of the particles, **v**_*i*_, and a friction coefficient, *γ*, whereas the random force is modeled using a Gaussian random variable *ζ* with zero mean and unit variance. *ω*^*R*^ = (1 − *r*_*ij*_∕*r*_*c*_) and ${\omega }^{D}={({\omega }^{R})}^{2}$ are weighting factors, and the amplitude parameter of the random force, *σ*, is related to the dissipative force through the fluctuation-dissipation theorem as

5 $${\sigma }^{2}=2\gamma {k}_{B}T.$$

Moreover, for the simulations reported herein, the beads belonging to a particular molecule are connected using harmonic springs, ${{\bf{F}}}_{ij}^{S}={k}_{s}({r}_{eq}-{r}_{ij})$ between two consecutive beads and a small bending potential, ${U}_{bend}={k}_{b}(1+\cos \theta )$ between three consecutive beads, where *k*_*s*_ = 400 is the spring constant and *r*_*eq*_ = 0.95 is the equilibrium bond length. Two values for the bond-bending constant are used here: *k*_*b*_ = 2, as used by Mohagheghi and Khomami^18^, and *k*_*b*_ = 2.38, which is a fine-tuned value for reproducing entangled polyethylene melt properties obtained from MD simulation, as discussed in the Results and Discussion section.

To prevent chain-crossing in DPD simulations of an entangled liquid, we employed a computationally efficient method developed by Nikunen *et al*.^42^. This method suggests that chain crossing can be avoided by tuning the conservative force and enforcing a simple geometric constraint. Mohagheghi and Khomami^18^ showed that the criterion could be met by choosing a proper level of coarse-graining and an appropriate spring constant. In the present work, we use the same level of coarse-graining and the parameter set as those used by Mohagheghi and Khomami^18^—see the cited reference for more details on the chain-crossing criteria. Table 1 summarizes the DPD model parameter numerical values and their relevant units. In this table, *m* is DPD particle mass, which is considered as the mass unit for the DPD model. In the present DPD simulations, the geometric chain-crossing criterion remained satisfied in all cases, regardless of whether *k*_*b*_ = 2 or 2.38.

**Table 1:** DPD simulations parameters.

Quantity	Value	Units
*a*	200	*k*_*B*_*T*/*r*_*c*_
*γ*	4.5	${(m{k}_{B}T/{r}_{c}^{2})}^{1/2}$
*σ*	3.0	${(m{({k}_{B}T)}^{3}/{r}_{c}^{2})}^{1/4}$
*k*_*s*_	400	${k}_{B}T/{r}_{c}^{2}$
*r*_*eq*_	0.95	*r*_*c*_
*k*_*b*_	2.38 (N233) 2.0 (N250 and N400)	*k*_*B*_*T*

Equilibrium and nonequilibrium DPD simulations of monodisperse linear polyethylene molecules composed of 233 (N233), 250 (N250), and 400 (N400) beads per chain were performed in the *NVT* ensemble at a constant reduced bead number density of *ρ*_*bead*_ = 1 and constant temperature of *k*_*B*_*T* = 1. Newton’s equations of motion were integrated using the Velocity-Verlet algorithm, implemented within the Large-scale Atomic/Molecular Massively Parallel Simulator (LAMMPS^43^) environment, to perform the DPD simulations. Nonequilibrium DPD simulations were performed using a boundary-driven approach along with the Lagrangian rhomboid periodic boundary conditions. The simulation time step was 0.012 for all the DPD simulations and the parameter sets for all systems were the same, except that the spring constant was set at *k*_*b*_ = 2.38 for N233 and *k*_*b*_ = 2 for N250 and N400—see Table 1. Table 2 summarizes the cell details for the DPD simulations.

**Table 2:** The DPD simulation cell characteristics. *L*_*x*_, *L*_*y*_, and *L*_*x*_ are cell lengths in *x*, *y*, and *z* dimensions, respectively, in reduced (*r*_*c*_) units.

System	*L*_*x*_	*L*_*y*_	*L*_*z*_	Number of particles
N233	97.8	41	41	164,265
N250	100	100	52.92	528,750
N400	130	65	65	549,600

Equilibrium and nonequilibrium molecular dynamics simulations of monodisperse, linear, C_700_H_1402_ and C_1000_H_2002_ melts were performed in the *NVT* ensemble at a constant density of 0.766 g/cm^3^ (corresponding to a pressure of 1 atm) and constant temperature of 450 K. The Siepmann-Karaboni-Smit (SKS) united-atom potential model^34^ was used to quantify the energetic interactions between the atomistic components of the polyethylene liquid except that the rigid bond between adjacent atoms in the original model was replaced with a harmonic potential function. The p-SLLOD equations of motion^44–48^ were used to perform the NEMD simulations, which were maintained at a constant temperature of 450 K using a Nosé-Hoover thermostat^49,50^. The equations were integrated using the reversible-Reference System Propagator Algorithm (r-RESPA)^51^ with two different time steps. The long timestep was 4.70 fs, which was used for the slowly varying nonbonded Lennard-Jones interactions, and the short timestep was 1.176 fs (one-fourth of the long timestep) for the rapidly varying forces including bond-bending, bond-stretching, and bond-torsional interactions. The set of p-SLLOD evolution equations for the particle positions and momenta were implemented and integrated within the LAMMPS^43^ environment. The MD simulation results are mostly based on prior work of Nafar Sefiddashti *et al*.^10,12,32,33^—see the cited references for the simulation details. Specifically, the simulation cell sizes and the number of particles can be found for the C_1000_H_2002_ liquid in Table 1 of ref. ^33^, and for the C_700_H_1402_ melt in ref. ^32^ (equilibrium and shear simulations) and ref. ^10^ (PEF simulation). A detailed discussion of the SKS model equations and parameters can be found elsewhere^9,12,52^.

## Results and Discussion

### Equilibrium simulations

DPD simulation parameters and outputs are usually expressed in reduced (dimensionless) units. The most common normal or natural units for DPD simulations are the bead’s mass, *m*_*bead*_, as the mass unit, the pairwise potential cut off distance, *r*_*c*_, as the length unit, and *k*_*B*_*T* as the energy unit (which could be used to define a time unit). As a consequence, DPD results can not be compared directly with the experimental results and other real-unit simulations, such as atomistic MD. Therefore, the development of simple and accurate rescaling parameters through adequate mapping of DPD results to corresponding MD results could lead to significant advantages in the realistic and reliable analysis of the dynamics of polymeric liquids using computationally affordable methods such as DPD.

It is worth noting that practically many MD simulations are also performed in reduced units, for instance, Lennard-Jones (LJ) units where the monomer mass, LJ distance parameter, and LJ energy parameter are assumed to be unity; However, since the values of these parameters are known in real units, all quantities can be equivalently expressed in real (*e.g*., SI) units, and hence easily compared against experimental results.

Comparing various coarse-grained simulations and developing scaling methods and parameters has been a subject of many recent studies^53–55^. For example, Masubuchi and Uneyama^54^ compared some results from a few multichain models, including multichain slip-spring and slip-link (PCN) models, with Kremer-Grest type coarse-grained MD simulations^56,57^ and proposed conversion parameters for the units of length, time, and bead number. However, as the Kremer-Grest type simulations are coarse-grained simulations themselves, a direct comparison with experimental data cannot be performed using these conversion parameters. In this work, we focus on finding scaling parameters for DPD simulations using united atom MD simulations (with real units), which are essential to paving the way for direct comparison of DPD simulation results and experiment.

**Figure 1:**
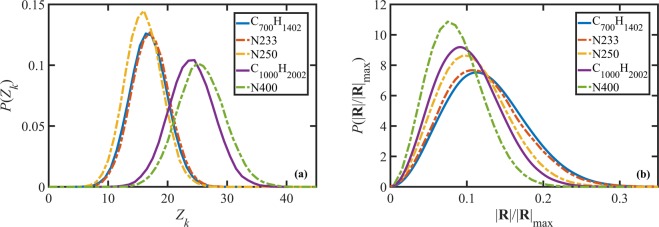
Probability distribution functions of the number of entanglements per chain (**a**) and normalized chain end-to-end distance (**b**) for C_700_H_1402_ and C_1000_H_2002_ liquids from MD simulations, and N233, N250, and N400 from DPD simulations under quiescent conditions.The bond-bending parameter was *k*_*b*_ = 2.38 for the N233, and *k*_*b*_ = 2 for N250 and N400 simulations.

As discussed in the previous section, chain-crossing in DPD could be avoided by choosing a proper level of coarse-graining and an appropriate spring constant^18^. For the selected parameter set and simulation conditions (*i.e*., temperature and density) discussed in the previous section, this proper level of coarse-graining for an entangled polyethylene melt is obtained by lumping roughly three methyl monomeric units into a single DPD bead. Moreover, a coarse-grained linear chain consisting of *N* beads should be expected to exhibit similar entanglement properties as those of a linear C_3N_H_6N+2_ polyethylene melt. This can be investigated by comparing the probability distribution functions (PDFs) for the entanglement densities and the number of Kuhn segments per chain obtained from DPD and MD simulations. Figure 1a displays the PDFs of the entanglement density, *Z*_*k*_, for C_700_H_1402_ and C_1000_H_2002_ melts from MD simulations and the N233, N250, and N400 liquids from DPD simulations under quiescent conditions obtained from Z1-code analysis^58^. The ratios between the number of monomeric units and the number of beads are 2.8 and 2.5 for the C_700_H_1402_/N250 and C_1000_H_2002_/N400 pairs, respectively. These ratios are slightly smaller than the ultimate value of 3 that will be shown to represent equivalent MD and DPD simulation pairs; however the deviation of these ratios from 3 is small enough to allow an insightful comparison of the DPD and MD results and to fine-tune the coarse-grained DPD model.

Figure 1a reveals that despite a good agreement between the PDFs for C_700_H_1402_/N250 and C_1000_H_2002_/N400 simulation pairs, they are not precisely equivalent. The discrepancy could be due to various factors including the degree of coarse-graining (bead ratio or ratio of Kuhn segments) and relative chain flexibility (controlled by *k*_*b*_ in the DPD model). The number of Kuhn segments per chain can be readily calculated from the simulation results as ${N}_{k}=| {\bf{R}}{| }_{\max }/\langle {R}^{2}\rangle$, assuming Gaussian statistics, where $| {\bf{R}}{| }_{\max }$ is a chain’s maximum contour length and ⟨*R*^2^⟩ is the ensemble average of the squared chain end-to-end magnitude. Table 3 shows that there is a significant discrepancy between the number of Kuhn segments for the C_700_H_1402_/N250 and C_1000_H_2002_/N400 simulation pairs. These discrepancies (in both *Z*_*k*_ and *N*_*k*_) suggest that both the coarse-graining ratio and bond-bending potential need to be optimized to map the DPD results accurately to a real physical (in the present case, polyethylene) liquid through comparison with MD simulations.

**Table 3:** Structural, dynamical, and topological properties of the simulated liquids under quiescent conditions.

Chain	*τ*_*d*_	${{\boldsymbol{\langle }}{{\boldsymbol{R}}}^{{\bf{2}}}{\boldsymbol{\rangle }}}^{{\bf{1}}{\boldsymbol{/}}{\bf{2}}}$	⟨*Z*_*k*_⟩	*N*_*k*_	*D*_*G*_
C_700_H_1402_	1216 ns	123.5 Å	17.5	53	3.22 × 10^−1^ Å^2^∕ns
N233	1.15 × 10^6^	29.1	16.9	57	1.97 × 10^−5^
N250	1.07 × 10^6^	27.9	16	72	1.6 × 10^−5^
C_1000_H_2002_	5270 ns	141.8 Å	24.3	83	1.28 × 10^−1^ Å^2^∕ns
N400	5.3 × 10^6^	35.2	26	116	6.1 × 10^−6^

This optimization was performed and the results are also presented in Fig. 1 as the N233 DPD simulation results. The optimized coarse-graining bead ratio and bending potential turned out to be 3 and *K*_*b*_ = 2.38, respectively. Figure 1b presents the probability distribution function for the chain end-to-end distance, ∣**R**∣, normalized with the chain contour length under quiescent conditions for the simulated liquids. The distributions are Gaussian, as expected, with a peak at an ensemble average normalized end-to-end distance, ${\langle {R}^{2}\rangle }^{1/2}$/$| {\bf{R}}{| }_{\max }$ for each liquid. Note that the peak position theoretically corresponds to ${N}_{k}^{-1/2}$ (${\langle {R}^{2}\rangle }^{1/2}$/$| {\bf{R}}{| }_{\max }={({N}_{k}{b}^{2})}^{1/2}$/$({N}_{k}b)={N}_{k}^{-1/2}$, where *b* is the Kuhn length). The apparent differences in Fig. 1a,b between the C_700_H_1402_-N250 and C_1000_H_2002_-N400 pairs is due to a suboptimal matching of the number of Kuhn segments resulting from a higher chain flexibility in the DPD simulations (which used *k*_*b*_ = 2). However, for the optimized parameter of *k*_*b*_ = 2.38 and a bead ratio of 3, the N233 DPD simulation results for both *Z*_*k*_ and $| {\bf{R}}| /| {\bf{R}}{| }_{\max }$ practically overlap with those of the corresponding MD simulation data of the C_700_H_1402_ melt.

For an entangled liquid, the ensemble average equilibrium chain end-to-end distance, ${\langle {R}^{2}\rangle }^{1/2}$, radius of gyration, ${\langle {R}_{g}^{2}\rangle }^{1/2}$, and the reptation (disengagement) time, *τ*_*d*_, are perhaps the most suitable candidates for scaling the length and time units of the DPD simulations based on MD simulation data. Prior DPD simulations have shown that the root-mean-squared end-to-end distance and disengagement time scale with the number of beads, *N*, as *N*^1∕2^ and *N*^3.3^, respectively^18^, in agreement with both experiment and the MD simulations^31–33^. Molecular Dynamics simulations and experiments also suggest that these quantities are physically reasonable scaling choices. On the other hand, these properties only reflect the long time (${\mathcal{O}}({\tau }_{d})$) dynamics of the system, and hence may not work properly at shorter time scales. Also, such scaling parameters that are obtained solely based on equilibrium properties are not guaranteed to be valid in a nonequilibrium flow application. These issues will be discussed later. Table 3 presents several equilibrium properties of the polyethylene liquids from both MD and DPD simulations. The disengagement time was calculated from the longest decorrelation time of the chain end-to-end vector autocorrelation function, as described in prior work^32,33^. A length scaling factor can now be postulated as

6 $${F}_{l}=\frac{{\langle {R}^{2}\rangle }_{MD}^{1/2}}{{\langle {R}^{2}\rangle }_{DPD}^{1/2}}.$$

Similarly, a time scaling factor is defined as

7 $${F}_{t}=\frac{{\tau }_{d}^{MD}}{{\tau }_{d}^{DPD}}.$$

To use these scaling factors, one should multiply the DPD length and time units by *F*_*l*_ and *F*_*t*_, respectively, to obtain their values in real units.

A scaling factor for the stress tensor, ***σ***, can be defined as the ratio of plateau modulus obtained from the MD and DPD simulations. Calculation of the plateau modulus, however, can be computationally expensive, especially for MD simulations. Alternatively, the zero-shear viscosity can be used to define a scaling factor for viscosity as

8 $${F}_{v}=\frac{{\eta }_{0}^{MD}}{{\eta }_{0}^{DPD}},$$

where ${\eta }_{0}^{MD}$ and ${\eta }_{0}^{DPD}$ are the zero-shear viscosities calculated from NEMD (extrapolated to zero strain rate) and DPD simulations. The zero-shear viscosity in both NEMD and DPD simulations scales as *N*^3.3^. Since all three scaling factors have numerators and denominators that scale with *N* equally (for entangled liquids), these ratios should not change with *N* (for a specific value of the MD/DPD particle ratio) in the long-chain limit (above *C*_*∞*_).

These three scaling factors constitute the most important principal physical dimensions, which can be employed to extract units (scaling factors) for other physical quantities, such as stress, velocity, etc., using a simple dimensional analysis. Scaling factors for the stress and normal stress coefficients can be readily calculated, respectively, as

9 $${F}_{s}=\frac{{\eta }_{0}^{MD}}{{\eta }_{0}^{DPD}{F}_{t}}=\frac{{F}_{v}}{{F}_{t}},$$

10 $${F}_{\Psi }=\frac{{\eta }_{0}^{MD}{F}_{t}}{{\eta }_{0}^{DPD}}={F}_{v}{F}_{t}.$$

It should be noted that the stress scaling factor from Eq. (9) should not be used for converting the thermodynamic pressure due to significant differences among the potential models employed in MD and DPD simulations. However, it works fairly well for scaling all components of the extra stress tensor, including shear and normal stresses. The mass conversion factor can be readily calculated as *F*_*m*_ = *F*_*ν*_*F*_*t*_*F*_*l*_, using a similar dimensional analysis. The numerical value of *F*_*m*_ in SI units for N233 is *F*_*m*_ = 2.022 × 10^−26^ kg. A conversion factor for density can then be calculated as *F*_*ρ*_ = *F*_*m*_/${F}_{l}^{3}=265.7$ kg/m^3^ for N233. Note that the mass density in DPD simulations is *ρ*_*m*_ = 1, as the DPD particle mass is *m* = 1. Hence, DPD mass density can be expressed in SI units as *ρ*_*m*_*F*_*ρ*_, that is, 1 × 265.7 = 265.7 kg/m^3^ for N233; in physical units of the PE melt, this corresponds to a density of 3 × 265.7 = 797.1 kg/m^3^, which is within 4% of the density value used in the MD simulations, *i.e*., 766 kg/m^3^. This ratio is not coincident; it arises from the level of coarse-graining used here, *i.e*., lumping three CH2 united atoms of MD into one DPD bead.

Table 4 collects the length, time, and viscosity scaling factors for the C_700_H_1402_/N233, C_700_H_1402_/N250, and C_1000_H_2002_/N400 melt systems. Note that the N250 and N400 systems have numerical values for the scaling factors that are relatively consistent with those of the optimized N233 system, except for the *F*_*v*_ scaling factor, which varies significantly from one system to another.

**Table 4:** Length, time, and viscosity scaling factors for the C_700_H_1402_/N233, C_700_H_1402_/N250, and C_1000_H_2002_/N400 melts.

Chain	*F*_*t*_ (ns)	*F*_*l*_ (Å)	*F*_*v*_ (Pa s)
N233	1.057 × 10^−3^	4.238	4.515 × 10^−5^
N250	1.136 × 10^−3^	4.427	6.332 × 10^−5^
N400	9.943 × 10^−4^	4.028	7.428 × 10^−5^

Armed with scaling factors for length and time, a theoretical scaling factor can be calculated for diffusivity. From a dimensional point of view, diffusivity is expressed in (length)^2^/time units. Hence a diffusivity scaling factor can be defined as ${F}_{d}={F}_{l}^{2}$/*F*_*t*_, and calculated as 1.699 × 10^4^ Å^2^/ns for N233, 1.724 × 10^4^ Å^2^/ns for N250, and 1.632 × 10^4^ Å^2^/ns for N400, based on the quantities displayed in Table 4. These values lead to estimations of 3.364 × 10^−1^ Å^2^/ns and 2.759 × 10^−1^ Å^2^/ns for the diffusivity of C_700_H_1402_ based on N233 and N250 results, respectively, and 1.0 × 10^−1^ Å^2^/ns for the diffusivity of C_1000_H_2002_ based on N400 results. These estimations differ by about 4% based on N233, 14% based on N250, and 9% percent based on N400, compared to the corresponding values from the MD simulations. The agreement between the estimated values and the MD values is very good, especially for the optimized N233 system ( ≈ 4%), considering the statistical error associated with estimating the diffusivity coefficient as well as the error in estimating the disengagement time, ensemble average chain end-to-end distance, and the corresponding length and time scaling factors.

Both the disengagement time and diffusivity are steady-state and large timescale properties of the system that only reflect the longer time dynamics (${\mathcal{O}}({\tau }_{d})$) of the liquids. It could be argued that any length and time scale obtained from these properties is not guaranteed to be valid for shorter time and length scales, and hence this issue needs to be examined under those conditions. Furthermore, the same skepticism exists for any and all transient physical properties that are scaled using these factors. Therefore, we next examine the segmental mean-squared displacement, $\phi (t)=\langle {({{\bf{r}}}_{n}(t+\tau )-{{\bf{r}}}_{n}(\tau ))}^{2}\rangle$ (**r**_*n*_ is the position vector of the *n*-th monomer or bead)^3^, which quantifies steady-state dynamics over a wide spectrum of time and length scales simultaneously, ranging from the entanglement time *τ*_*e*_ and tube diameter *a*, to the disengagement time *τ*_*d*_ and chain end-to-end distance ∣**R**∣. Hence, comparison of *ϕ* between the various simulation methods is instructive for examining the accuracy of the simulations as well as the validity of the scaling factors used for mapping their results. Reptation theory predicts four regimes for the motion of chain segments^3^. For times shorter than *τ*_*e*_, the segmental MSD scales as *t*^1∕2^. For *τ*_*e*_ < *t* < *τ*_*R*_ (where *τ*_*R*_ is the Rouse relaxation time), the chain motion is affected by the Rouse-like diffusive motion (and the tube constraints) and the segmental MSD scales as *t*^1∕4^. For *τ*_*R*_ < *t* < *τ*_*d*_, *ϕ* scales as *t*^1∕2^, and for *t* > *τ*_*d*_, reptation becomes the dominant diffusive mechanism wherein *ϕ* scales as *t*^1^.

**Figure 2:**
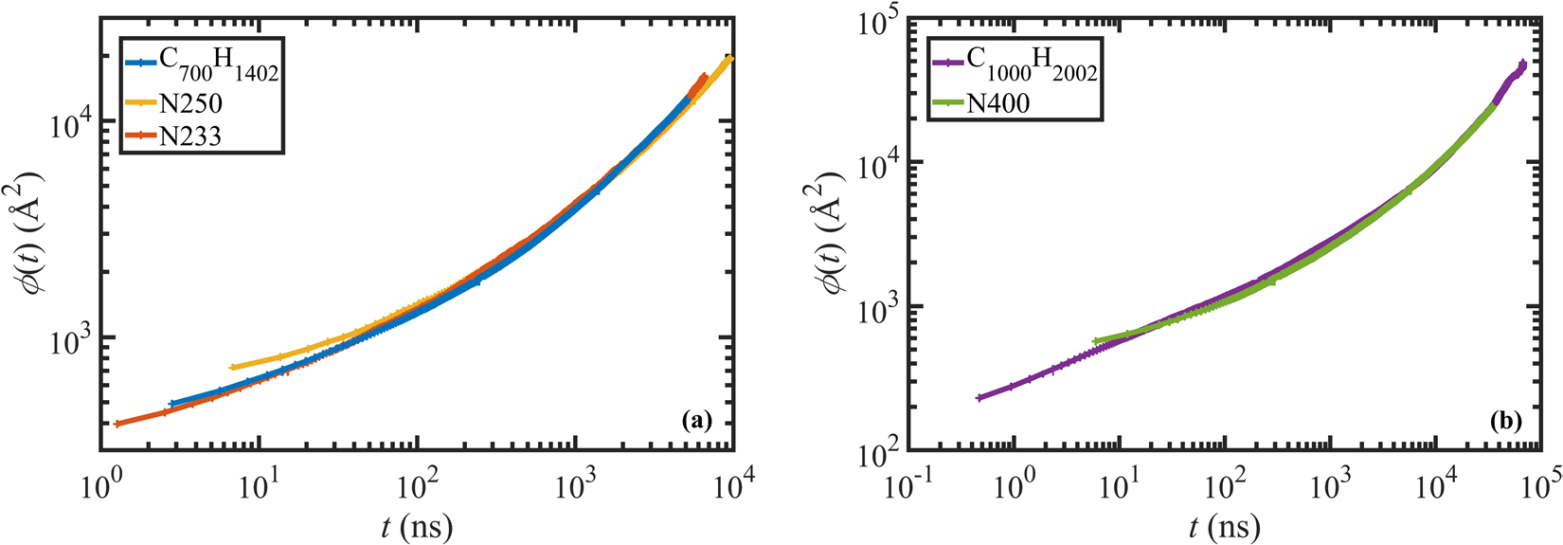
Segmental mean-squared displacement versus time of the 50% centermost chain monomeric units for C_700_H_1402_, N233, and N250 (panel (a)), and C_1000_H_2002_ and N400 (panel (b)). The MD data were taken from refs. ^32,33^ and the DPD data were scaled using *F*_*d*_ and *F*_*t*_ from Table 4.

Figure 2 displays the segmental mean-squared displacement, *ϕ*, as a function of time for the C_700_H_1402_, N233, and N250 (panel (a)) and the C_1000_H_2002_ and N400 (panel (b)) liquids. The DPD data for N233, N250, and N400 were scaled using *F*_*d*_ and *F*_*t*_ from Table 4, as explained above. It is evident from this figure that a single scaling factor works satisfactorily over the entire range of time and length scales. Note that both plots cover times from 5 ns (*i.e*., roughly the entanglement time of polyethylene^31–33,52^) to times well above the disengagement time for each liquid. Furthermore, the good agreement between the DPD and MD results (especially for the C_700_H_1402_/N233 pair) confirms that the proposed DPD method is capable of capturing the short and long time-length scale dynamics reliably. This is not surprising as the smallest timescale of the DPD simulation remains well below the entanglement time of the macromolecular system (or, equivalently, the shortest DPD length scale remains small relative to the tube diameter).

### Nonequilibrium simulations

It has been demonstrated thus far that simple time and length scaling factors obtained from long-time properties, such as the overall chain dimensions and the reptation time, can be used for reliably mapping the steady-state properties calculated from DPD simulations to real, physical dimensions; however, the same demonstration needs to be performed under transient conditions and in nonequilibrium situations. In this section, we examine transient and steady-state flow behavior for several different systems: the C_700_H_1402_/N233 pair subject to simple shear flow at *Wi* = 50 and at *Wi* = 1000, the C_1000_H_2002_/N400 pair subject to simple shear flow at *Wi* = 58, and the C_700_H_1402_/N250 pair subject to planar elongational flow (PEF) at *Wi*_*R*_ ≈ 1.6 (*Wi* = 30). The reptation Weissenberg number, $Wi\equiv \dot{\gamma }{\tau }_{d}$, is proportional to the shear rate, $\dot{\gamma }$, which is made dimensionless using the disengagement time of the liquid, and the Rouse Weissenberg number, $W{i}_{R}\equiv \dot{\varepsilon }{\tau }_{R}$, is proportional to the extension rate, $\dot{\varepsilon }$, which is made dimensionless using the Rouse relaxation time of the liquid. It should be noted that in all cases the strain rates are above ${\tau }_{R}^{-1}$ of the liquids, and hence flows are highly nonlinear. Indeed, we chose the values *Wi* = 50 and *Wi* = 1000 because the former is greater than ${\tau }_{R}^{-1}$ and the latter is greater than ${\tau }_{e}^{-1}$, thus covering two very different but highly nonlinear flow regimes. At such high flow strength, most of the liquids’ structural and topological properties are significantly perturbed as compared to their properties under quiescent conditions. It is logical to assume that, if the scaling methods are reliable under quiescent conditions and at such high flow strengths, they should also perform reasonably well over a wide range of flow strengths including the linear, nonlinear, and highly nonlinear flow regimes. The velocity gradient tensor assumes the form

11 $$\nabla {\bf{u}}=\left(\begin{array}{lll}0 & 0 & 0\\ \dot{\gamma } & 0 & 0\\ 0 & 0 & 0\end{array}\right)$$

for the simple shear flow and

12 $$\nabla {\bf{u}}=\left(\begin{array}{lll}\dot{\varepsilon } & 0 & 0\\ 0 & -\dot{\varepsilon } & 0\\ 0 & 0 & 0\end{array}\right)$$

for planar elongational flow.

**Figure 3:**
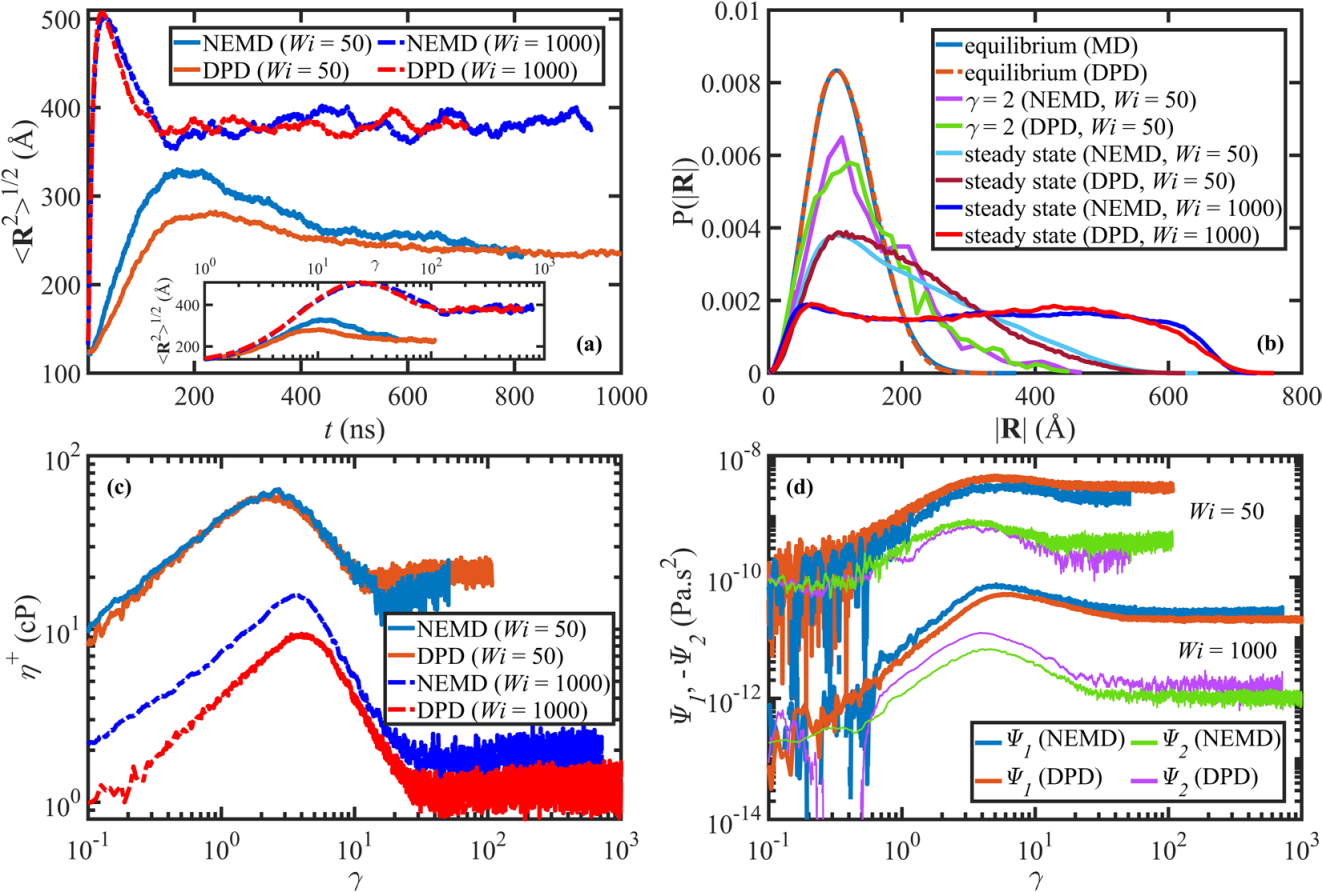
(**a**) The ensemble average chain end-to-end distance as a function of time and shear strain (inset) upon startup of shear flow for the C_700_H_1402_ and N233 liquids. (**b**) The probability distribution functions for the chain end-to-end distance at equilibrium, *ε*_*H*_ = 2, and steady-state for the same liquids. The transient shear viscosity (**c**) and normal stress coefficients (**d**) as functions of shear strain. The four top curves in panel (d) represent *Wi* = 50 and the four bottom curves represent *Wi* = 1000 simulations. The NEMD data of panel (c) for *Wi* = 1000 were taken from ref. ^59^ and the DPD data were scaled as explained in the text.

Figure 3a displays the evolution of the ensemble average chain end-to-end distance as a function of time for the C_700_H_1402_ and N233 liquids upon startup of shear flow at *Wi* = 50 and *Wi* = 1000. The DPD length unit has been scaled using the end-to-end distances of the NEMD and DPD simulations under equilibrium conditions (according to Eq. (6): *F*_*l*_ = 4.238 from Table 4) and the time unit is scaled using *F*_*t*_ from Table 4. Note that at the initial instant, both simulations occupy microstates that are statisitically not at the quiescent ensenmble average microstate; however, the scaling method was applied using the equilibrium average results represented in Table 4 rather than employing a value of *F*_*l*_ based on the NEMD and DPD systems’ instantaneous microstructural states at the initial time (*t* = 0). In general, there is a good agreement between the two simulations, suggesting that the proposed scaling method suffices at least for mapping preaveraged quantities such as the ensemble average molecular extension over a wide rage of flow strength. However, the average extension overshoot is slightly underpredicted by DPD for *Wi* = 50. This could be because *k*_*b*_ was optimized under quiescent conditions, whereas the DPD chains could possibly be stiffer than the NEMD chains (which follow the SKS potential) at the overshoot time. Nevertheless, the overshoot time is captured correctly by the scaled DPD data. The inset of Fig. 3a shows the same quantity as a function of shear strain, $\gamma =\dot{\gamma }t$, which provides a quantitative measure of the relative deformation of the material within the applied shear flow field. Note that *γ* also serves as a dimensionless time, which is not affected by the time scaling method used here. The good agreement between the curves in the inset suggests that the length scaling works fairly well under transient conditions. Overall, it is evident from Fig. 3a that both the proposed length and time scaling factors independently perform well under transient conditions.

The probability distribution functions of the chain end-to-end distance are presented in Fig. 3b at equilibrium and various shear strains/rates during the evolution of the systems. The distributions are Gaussian and practically identical at equilibrium, as evident from Fig. 3b. The DPD end-to-end distances in this figure are scaled using *F*_*l*_ = 4.238 from Table 4. As flow begins, the molecules become partially oriented and stretched, and the distributions widen and become non-Gaussian—see the *γ* = 2 and steady-state curves in Fig. 3b. Note that *γ* = 2 corresponds to the stress overshoot time for *Wi* = 50 (see Fig. 3c). The quantitative agreement between these curves demonstrates that the DPD simulations and the proposed length scaling method reproduce the NEMD results not only on the mean-field level (*e.g*., average chain extension), but also on the molecular level.

The transient shear viscosity (${\eta }^{+}\equiv -{\sigma }_{xy}/\dot{\gamma }$) is presented in Fig. 3c for the C_700_H_1402_ and N233 liquids upon startup of shear flow at *Wi* = 50 and *Wi* = 1000. The DPD values were scaled using *F*_*v*_ = 4.515 × 10^−5^ Pa.s, as obtained from the ratio of the shear viscosities of C_700_H_1402_ (taken from ref. ^32^) and N233 at *Wi* = 1. The first (${\Psi }_{1}\equiv ({\sigma }_{xx}-{\sigma }_{yy})/{\dot{\gamma }}^{2}$) and second (${\Psi }_{2}\equiv ({\sigma }_{yy}-{\sigma }_{zz})/{\dot{\gamma }}^{2}$) normal stress coefficients as functions of shear strain are displayed in Fig. 3d. The DPD values were scaled using *F*_*Ψ*_ = 6.6958 × 10^−17^ Pa.s^2^, according to Eq. (10). The agreement between the NEMD and scaled DPD values for all these rheological functions is nearly quantitative for both *Wi*’s, except for the *η*^+^ and *Ψ*_2_ overshoots at *Wi* = 1000. It is not apparent whether this minor discrepancy is due to the viscosity scaling procedure or the DPD simulation potentials. It should be noted that *F*_*Ψ*_ is subject to two error sources since it is calculated as the product of two other factors (*i.e*., *F*_*v*_ and *F*_*t*_), which are themselves prone to error. Nevertheless, the overshoot strains are captured by the DPD simulation fairly well in both cases.

**Figure 4:**
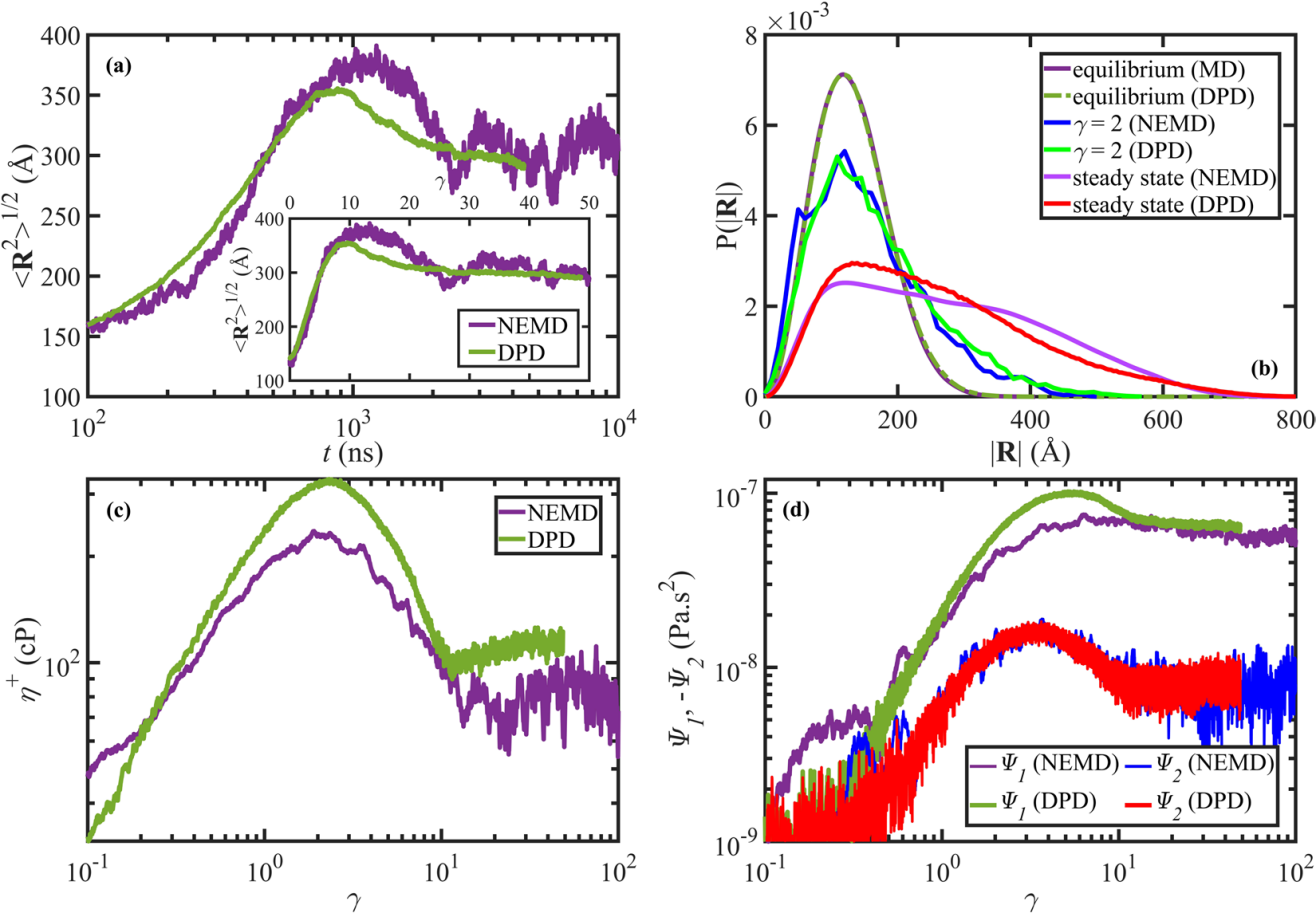
(**a**) The ensemble average chain end-to-end distance as a function of time (main panel) and shear strain (inset) upon startup of shear flow at *Wi* = 58 for the C_1000_H_2002_ and N400 liquids. (**b**) The probability distribution functions for the chain end-to-end distance at equilibrium, *ε*_*H*_ = 2, and steady-state for the same liquids. The shear viscosity (**c**) and normal stress coefficients (**d**) as functions of shear strain. The NEMD data were partially taken from ref. ^33^ and the DPD data were scaled as explained in the text.

Figure 4 is the counterpart of Fig. 3 for the startup shear simulations of the C_1000_H_2002_ and N400 melts at *Wi* = 58. The reasonable agreement between the evolution of the ensemble average end-to-end distance between the NEMD and DPD simulation data, as displayed in Fig. 4a, verifies again that both the length and time scaling factors work fairly well under transient conditions of startup shear flow. The inset of Fig. 4a shows the end-to-end distance as a function of shear strain, *γ*. Similar to the C_700_H_1402_/N233 case (see 3a), there is minor discrepancy between the NEMD and scaled DPD data after the overshoot in Fig. 4a. In addition to previous comments concerning the C_700_H_1402_/N233 melt, the discrepancy here could also be due to the fact that the C_1000_H_2002_ and N400 systems are not identical, as discussed in the previous section; however, the end-to-end distance probability distribution functions at equilibrium are almost identical, as presented in Fig. 4b. Note that the DPD end-to-end distances in Fig. 4b are scaled using *F*_*d*_ = 4.028 from Table 4. The distributions at *γ* = 2, corresponding to the stress overshoot time for both simulations (see Fig. 4c), agree fairly well over all chain end-to-end distances.

The rheological material functions also agree reasonably between the NEMD and scaled DPD results, as demonstrated in Fig. 4c,d. The DPD values for the shear viscosity are scaled using *F*_*v*_ = 7.428 × 10^−5^ Pa s, as obtained from the ratio of the shear viscosities of the C_1000_H_2002_ and N400 melts at *Wi* = 1, taken from refs. ^33^ and^19^. The DPD values for the normal stress coefficients are scaled using *F*_*Ψ*_ = 7.8543 × 10^−17^ Pa s^2^, according to Eq. (10). Despite the reasonable quantitative agreement between the data at low shear strains, *i.e*., *γ* < 1, and high shear strain, *i.e*., *γ* > 10 (nearly steady-state), the agreement is only qualitative for *η*^+^ and *ψ*_1_ within the intermediate range of shear strains that includes most of the transient region. Specifically, there is a maximum of 45% difference between the shear viscosity values, which occurs at the shear stress overshoot time. Such a discrepancy could arise because of (at least) two reasons. One reason is that such a simple viscosity (stress) scaling method using the ratio of zero-shear viscosities, is not adequate for startup of shear flow, especially near the stress overshoot time. The other possible reason is that the C_1000_H_2002_ and N400 liquids are not precisely equivalent systems, as discussed above. In fact, the N400 melt is closer to a C_1200_H_2402_ polyethylene melt, whose molecular weight is 20% heavier than C_1000_H_2002_. It is worth emphasizing that viscosity scales as *M*^3.3^ under equilibrium conditions. Hence, a relatively small difference in polymer molecular weight could lead to a relatively large difference in viscosity. Also, the bond-bending potential parameter for the N400 simulation was taken as *k*_*b*_ = 2.0, which is slightly softer than the optimized potential; this could also lead to deviation of DPD results from those of NEMD simulations. Nevertheless, the overshoot strains are captured well by the DPD simulations, suggesting that the proposed scaling methods are fairly robust with respect to small inaccuracies in simulation parameters. Bear in mind that Fig. 4 was produced via simulations that did not employ the optimal parameters (*i.e*., *k*_*b*_ = 2.0 rather than the optimized value of 2.38 and bead ratio of 2.5 rather than the optimized value of 3), which also demonstrates the robustness of the proposed scaling factors.

**Figure 5:**
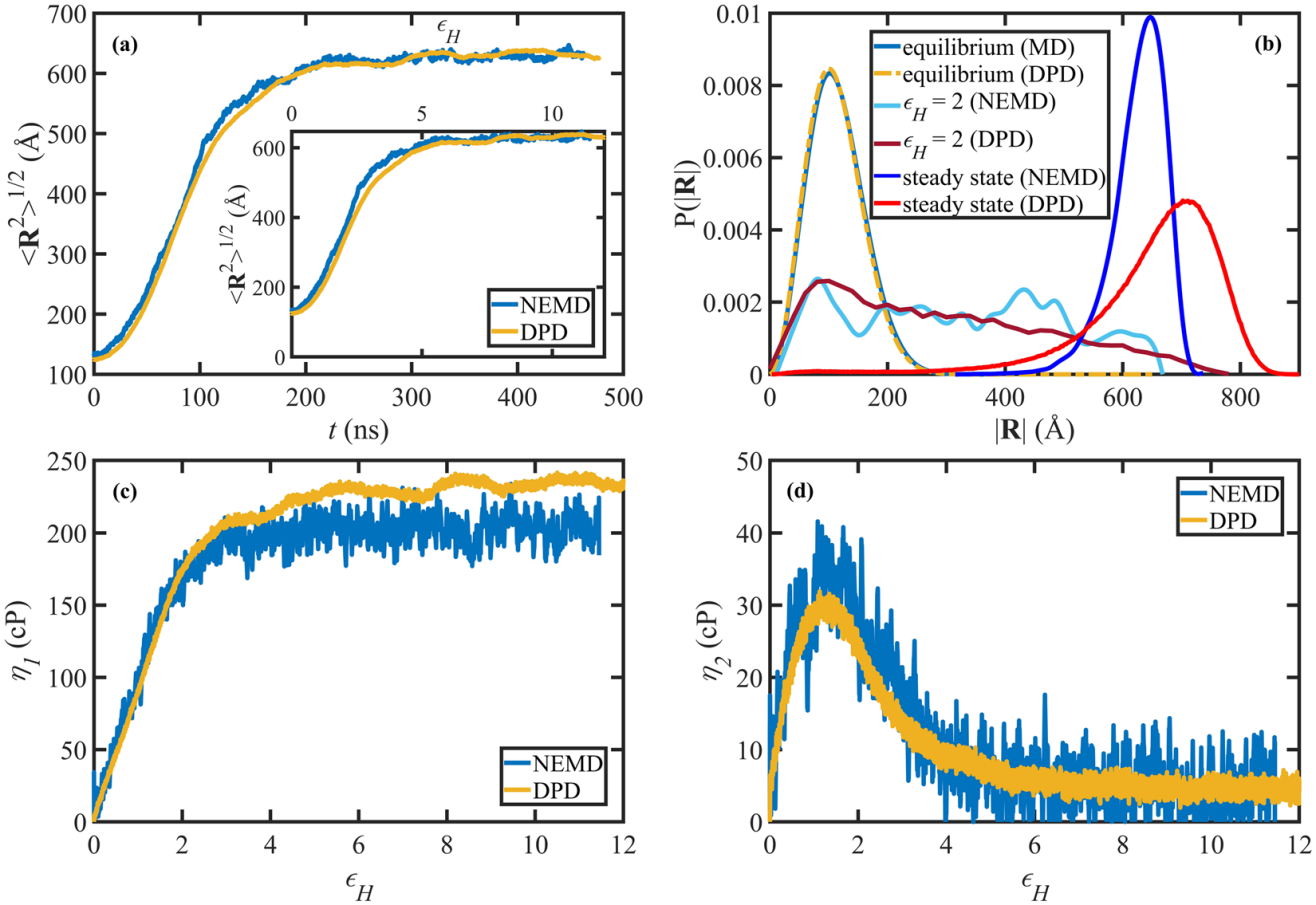
(**a**) The ensemble average chain end-to-end distance as a function of time (main panel) and Hencky strain (inset) upon startup of PEF at *Wi*_*R*_ = 1.6 for the C_700_H_1402_ and N250 liquids. (**b**) The probability distribution functions for the chain end-to-end distance at equilibrium, *ϵ*_*H*_ = 2, and steady-state for the same liquids. Also displayed are the primary, *η*_1_ (**c**), and secondary, *η*_2_ (**d**), extensional viscosities as functions of Hencky strain for the same liquids. The NEMD data of panel (a) were taken from ref. ^10^ and the DPD data were scaled as explained in the text.

For completeness, we examine the performance of the proposed DPD and scaling methods for another important type of flow, *i.e*., elongational flow. Elongational flows apply very large deformations which can stretch the molecules significantly as compared to quiscent conditions and shear flows. Hence, entangled polymeric liquid responses under elongational flows are commonly quite different from their responses under shear and require independent investigation since shear behavior is not necessarily generalizable to elongational conditions. Figure 5a displays the evolution of the ensemble average chain end-to-end distance as a function of time for the C_700_H_1402_ and N250 liquids upon startup of planar elongational flow at *Wi*_*R*_ = 1.6. The DPD length unit was scaled using Eq.(6) and *F*_*l*_ = 4.427 Å from Table 4, and the time unit was scaled using Eq. (7) and *F*_*t*_ = 1.136 × 10^−3^ from Table 4. There is a good agreement between the two simulations, suggesting that the proposed scaling method sufficiently matches averaged quantities, such as the ensemble average molecular extension under elongational conditions. The inset shows the same quantity as a function of Hencky strain, ${\varepsilon }_{H}=\dot{\varepsilon }t$, which provides a quantitative measure of the relative deformation of the material. Similarly to the shear strain, *ε*_*H*_ can be thought of as a dimensionless time, which allows comparing the respective time evolution behavior regardless of the time scaling. The good agreement between the curves in the inset of Fig. 5a implies that the length scaling works very well under transient conditions. Overall, it is evident from Fig. 5a that both the proposed length and time scaling factors independently perform well under transient elongational flow conditions.

The probability distribution functions of the chain end-to-end distance are presented in Fig. 5b at equilibrium, startup of PEF, and steady-state PEF conditions at *Wi*_*R*_ = 1.6 for the C_700_H_1402_ and N250 liquids. The distributions are Gaussian and practically identical at equilibrium, as evident from Fig. 5b. At *ε*_*H*_ = 2, although the distributions do not overlap, they exhibit some qualitatively similar features. Specifically, both distributions have a peak around 100 Å representing the coil configurations that have not yet been significantly disturbed by the imposed flow. Also, the probabilities for highly stretched configurations are significantly lower than those of the coiled and mildly stretched configurations for both the NEMD and DPD simulations. The differences between the distributions at *ε*_*H*_ = 2 could arise from various factors; however, most importantly, it should probably be attributed to the simulation cell size effect of the NEMD simulation rather than the scaling of the DPD length units. It has previously been shown that there could be significant cell size effects in elongational flow simulations of entangled liquids, especially in the calculation of the probability distribution function of chain extension^12^; however, such effects are not expected to influence the qualitative features exhibited by the liquid. See the Supplementary Materials of ref. ^12^ for more details. Meanwhile, the small NEMD simulation cell size translates to poor statistics and consequently large fluctuations in the transient PDF curve, which makes the resulting comparison inconclusive. Also, recall that the N250 melt is not quite equivalent to the C_700_H_1402_ liquid as both the employed bond-bending potential (*k*_*b*_ = 2) and the bead ratio (700/250 = 2.8) were slightly different than the optimized values of *k*_*b*_ = 2.38 and bead ratio (3).

Similar statements can be made when comparing the end-to-end distance distributions under steady-state conditions. Once again, the NEMD and DPD distributions do not completely match at steady state; however, they exhibit qualitatively similar behavior. Both distributions have peaks at high values of ∣**R**∣, corresponding to highly stretched molecules. The NEMD peak position is at approximately 650 Å, which is somewhat lower than that of the DPD peak at 710 Å. The NEMD distribution is Gaussian-like and relatively narrow, whereas the DPD distribution is comparatively wide (hence shorter than that of NEMD) with a long tail on the left side of the peak. Again, it appears that mostly quantitative differences between the distributions arise from cell-size effects in the NEMD simulations rather than the scaling factors or scaling methodology. In other words, it appears that the discrepancy arises from the fact that the NEMD simulation ensemble is not large enough to capture all the possible system configurational microstates and hence has a bias towards stretched configurations in agreement with observations of Nafar Sefidashti *et al*.^12^. It is worth noting that the NEMD simulation box contains 104 chains, which is significantly lower than the 2115 chains within the DPD simulation cell.

Figure 5c,d show the primary extensional viscosity, $${\eta }_{1}^{+}$$$$\equiv ({\sigma }_{xx}-{\sigma }_{yy})/(4\dot{\varepsilon })$$, and the secondary extensional viscosity, $${\eta }_{2}^{+}$$$$\equiv -({\sigma }_{yy}-{\sigma }_{zz})/(4\dot{\varepsilon })$$, respectively, as functions of Hencky strain upon startup of extensional flow at *Wi*_*R*_ = 1.6 for the C_700_H_1402_ and N250 simulations. The DPD data have been scaled using *F*_*v*_ = 6.332 × 10^−5^ Pa s, which is the ratio of the shear viscosities of the C_700_H_1402_ and N250 melts at *Wi* ≈ 1, taken from refs. ^32^ and^19^. It should be noted that we did not use the viscosity values at a lower *Wi* (*e.g*., *Wi* = 0.1) since those values are subject to huge statistical errors–see the error bars in Fig. 3b of ref. ^32^ and Fig. 1a of ref. ^19^. It is evident from Fig. 5c,d that both the scaled extensional viscosities from the DPD simulation agree very well with those of the NEMD simulation within statistical error bounds, implying the effective performance of the scaling method under both transient and steady-state conditions. These results again confirm that preaveraged rheological and microstructural quantities are not very sensitive to the proposed scaling method, nor are they very sensitive to the DPD simulation parameters per se.

## Conclusions

A simple and accurate method was developed to rescale equilibrium and nonequilibrium DPD simulation data, which are naturally dimensionless, to physical units that are consistent with those obtained from equivalent atomistic MD simulations of model linear entangled polyethylene liquids. The ratio of monomeric (MD) units to DPD particles was optimized at 3 and the bond-bending constant of the DPD simulations was optimized at *k*_*b*_ = 2.38. Three fundamental scaling factors were defined for length, time, and viscosity (equivalently, stress and force) as the ratios of relevant quantities that are readily obtained from MD and DPD simulations. Specifically, the length scaling factor was obtained from the ratio of the ensemble average chain end-to-end distances under quiescent conditions, the time scaling factor was calculated as the ratio of the disengagement times, and the viscosity scaling factor was obtained from the ratios of zero-shear viscosities. Most other scaling factors could be readily obtained from the fundamental ones using straightforward dimensional analysis. The numerical values for the optimized scaling factors were *F*_*t*_ = 1.057 × 10^−3^ ns for time, *F*_*l*_ = 4.238 Å for length, and *F*_*v*_ = 4.515 × 10^−5^ Pa s for viscosity. The comparisons of equilibrium MD and DPD simulation data, as well as nonequilibrium simulations including shear and planar extensional flows at high flow strengths, revealed that these simple scaling factors were capable of mapping the DPD data onto those of equivalent MD simulations in both transient and steady-state flows within the linear and nonlinear viscoelastic flow regimes. Since these scalings were developed for chain lengths above the long-chain characteristic ratio, *C*_*∞*_, they possibly apply to all polyethylene liquids of higher molecular weight. Because of its much greater computational efficiency, this new DPD model allows for simulations of polyethylene liquids of much greater molecular weight over longer time durations and with larger simulation cells than previously possible.

## Data Availability

The datasets generated and analyzed during the current study are available from the corresponding authors on reasonable request.
